# Effect of solar eclipse on microbes

**DOI:** 10.4103/0975-7406.76498

**Published:** 2011

**Authors:** Amrita Shriyan, Angri M. Bhat, Narendra Nayak

**Affiliations:** Department of Microbiology, A. J. Institute of Medical Sciences, Kuntikan, NH-17, Mangalore -575 004, Karnataka, India

**Keywords:** Antibiotic susceptibility pattern, bacteria, genotypic changes, phenotypic changes, solar eclipse, short waves, yeast

## Abstract

**Objective:**

A solar eclipse was observed in India on 15^th^ January, 2010. It was a total eclipse in some parts of the country, while it was a partial eclipse in other parts. Microorganisms play an important role in various phenomena on the earth. This study was undertaken to know the influence of solar eclipse on nature indirectly, by analyzing certain genotypic and phenotypic variations in prokaryotes and eukaryotes. Since yeast have similar gene expression as that of humans, investigations were pursued on Candida albicans. Hence the study of the effect of solar eclipse on cultures of *Staphylococcus aureus, Klebsiella species, Escherichia coli*,and *C. albicans* was performed in the laboratory. The effect of the total or partial eclipse on the microorganism isolated from clinical isolates was investigated during the time period from 11.15 am to 3.15 pm.

**Materials and Methods:**

Cultures of *S. aureus, Klebsiella* species, and *E. coli* colonies on nutrient agar slants and broth and *C. albicans* on Sabouraud’s dextrose agar plates and broth. Slants were exposed to sunlight during eclipse and exposure to normal sunlight at Mangalore, Dakshina Kannada district, Karnataka state, India.

**Results:**

There was significant change observed during exposure to normal sunlight and eclipse phase. Bacterial colonies showed difference in morphology on smear examination and sensitivity pattern during this study. One fungal species and three bacterial isolates were studied and changes were recorded. Fungal species showed a definite change in their morphology on exposure to sunlight during eclipse observed by stained smear examination from broth, plate, and slant.

**Conclusion:**

Present study concludes that blocking of the sun rays during eclipse does not harm prokaryotes and eukaryotes, instead promoted the progeny of predators in the race of better acclimatization and survival in the natural and changing environmental conditions.

Eclipse was observed in India on 15^th^ January, 2010. The solar eclipse of this day was an annular eclipse of the sun with a magnitude of 0.9190. A solar eclipse occurs when the moon passes between earth and the sun, thereby totally or partially obscuring earth’s view of the sun. An annular solar eclipse occurs when the moon’s apparent diameter is smaller than the sun, causing the sun to look like an annulus/ring, blocking most of the sun’s light. An annular eclipse appears as a partial eclipse over a region thousands of kilometers wide. At approximately 13:20 IST, the annular solar eclipse entered India at Thiruvananthapuram, Kerala, and exited India at Rameswaram, Tamil Nadu.

The eclipse was viewable for 10.4 min in India. It was a total eclipse in some parts of the country, while it was partial in other parts. This occurs near a new moon when the moon is between sun in some parts of the world. During this period, the direct sun rays do not fall on the earth; a significant trait of sun rays only falls which have an impact on the nature. To understand this, a primitive study is carried on a selected group of bacteria and *Candida albicans*.

The aim of this study was to scientifically prove life supportive influence of solar eclipse on prokaryotes and eukaryotes. To study the effect of solar eclipse, we selected bacteria for prokaryotes and *C.albicans* for eukaryotes. This subject is important to understand the concept that confluent growth on culture plate, morphological change, antigenic variation, decreased antibiotic sensitivity, and increased virulence mechanism is exhibited on exposure to solar eclipse by bacterial and yeast cultures. Phenotypic transitional variation is considered as a potential virulence character. This common feature observed in fungus refers to phenotypic switching which may assist transition from commensalisms to pathogenic phase of microorganisms.[1] The filament-inducing property of *C. albicans* was also studied by other workers as documented in our study.

Microbes invade the host defense mechanism. A phase transition in microbes explains drift and shift in the antigenicity of the microbes.[2] This change results in the incompatibility of the host defense mechanism, further leading to impairment of immunological status in altered occasions. With regard to C. *albicans* which form the germ tube, transitional stage of hyphal development is analogy to bacterial phase transition phenomenon. This factor has an influence on immunoregulatory of fungal surface components.[3–5] There was a markable metabolic activity observed before the emergence of germ tube. When the cell-wall deposit becomes polarized, the germ tube forms.[6] Phospholipases concentrated at the hyphal tip enhances virulence. Hyphae being larger than the yeast is more resistant to phagocytosis.[7]

## Materials and Methods

The study was carried out in A.J. Institute of Medical Sciences, Department of Microbiology, Mangalore, Karnataka, India.

Study of the effect of solar eclipse on *Staphylococcus aureus, Klebsiella* species, and *Escherichia coli* (cultures) and C. *albicans* culture were included in the study. The criterion adopted in this study was the demonstration of morphological changes observed during exposure to normal sunlight and eclipse phase. A detailed cultural characteristics and biochemical reactions was also compared. We alsoevaluated differences in antibiotic sensitivity of microorganisms in comparison with bacterialcultures at the time of exposure to solar eclipse and normal sunlight.

Bacterial and yeast colonies were inoculated onto brain heart infusion broth from Hi-Media Laboratories Pvt. Limited, Mumbai. These bacterial suspensionswere assayed during the time period from 11.15 am to 3.15 pm (total duration = 4 h).. Bacterial and yeast colonies were studied on nutrient agar, Nutrient broth and Sabourauds dextrose agar and broth, respectively.

Nutrient broth : Beef extract 1.50 g/l, yeast extract 1.50 g/l, peptic digest of animal tissue 5.0 g/l, sodium chloride 5.0 g/l, distilled water 1000 ml, pH to 7.5-7.6 from Hi-Media Laboratories Pvt. Limited, Mumbai.

Nutrient agar : To the ingredients as in nutrient broth (beef extract 1.50 g/l, yeast extract 1.50 g/l, peptic digest of animal tissue 5.0 g/l, sodium chloride 5.0 g/l, distilled water 1000 ml, pH to 7.5-7.6), add 15 g agar per liter. Dissolve the agar in nutrient broth and sterilize by autoclaving at 121° C for 15 min. Blood agar: Nutrient agar 100 ml, sheep blood (defibrinated) 10 ml from Hi-Media Laboratories Pvt. Limited, Mumbai. Mac Conkey’s agar : Peptic digest of animal tissue 20 g/l, lactose 10 g/l, sodium taurocholate 5g/l, neutral red 0.04 g/l, agar 20 g/l, Ph = 7.4±0.2 from Hi-Media Laboratories Pvt. Limited, Mumbai.

Sabourauds dextrose agar: Mycological peptone 10 g/l, dextrose 40 g/l, agar 15 g/l from Hi-Media Laboratories Pvt. Limited, Mumbai.

Muller Hinton agar(MHA): Beef infusion form 300 g/l, casein acid hydrolysate 17.50 g/l, starch 1.5 g/l, agar 17.0 g/l from Hi-Media Laboratories Pvt. Limited, Mumbai.

Hi-CHROME *Candida* differential agar: Peptone 15 g/l, yeast extract 4 g/l, dipotassium hydrogen phosphate 1 g/l, chromogenic mixture 7.22 g/l, chloramphenicol 0.50 g/l, agar 15 g/l, Ph 6.3±0.2 at 25 c from Hi-Media Laboratories Pvt. Limited, Mumbai.

*S. aureus, Klebsiella* species, and *E. coli* and C. *albicans* were studied after incubation for 24 h at 37 °C. It was further streaked on to blood agar and CHROM agar and incubated for 24 h. An isolated colony was picked aseptically fromthe agar plate and inoculated onto Muller Hinton broth and peptone water broth for further biochemical reactions and to perform antibiotic sensitivity testing. Antibiotic discs from Hi-Media Laboratories Pvt. Limited, Mumbai, were used for antibiotic sensitivity testing. *S. aureus, Klebsiella* species, and *E. coli*, and *C. albicans* were studied after incubation for 24 h at 37°C.

## Results

The study was performed during the time period from 11.15 am to 3.15 pm on 15 ^th^ January, 2010, at A.J. Institute of Medical Sciences and Research Centre in Mangalore, Dakshina Kannada District, Karnataka State. Number of cultures tested were cultures of *S. aureus, Klebsiella* species, *E. coli*,and C. *albicans* from clinical isolates. Interpretation of results was based on definite changes in their morphology on smear examination, cultural characteristics on blood agar, Mac Conkeys agar and nutrient agar, biochemical reactions: catalase test, indole test, coagulase test, methyl red test, vogues Prausker test, triple sugar iron agar, urease test, citrate utilization test, and antibiotic sensitivity pattern on Muller Hinton Agar by Kirby Bauer’s disc diffusion method.

### Effect of normal sunlight and solar eclipse on bacterial and yeast colonies

There was an appreciable difference in the morphology observed on gram-stained smears of microorganisms - *S. aureus, Klebsiella* species, and *E. coli*, and C. *albicans* cultures both in broth and agar media. While *S. aureus, Klebsiella* species, and *E. coli* were stained intensely/dark stained from those cultures which were exposed to normal sunlight. Uneven staining was observed in cultures exposed during eclipse in bacteria and *C. albicans* culture. When stained showed abundant formation of germ tubes with reduced cell size and densely stained nucleus and clear cytoplasm.[2–4,8]

The number of germ tube formation in *C. albicans* remarkably increased suggesting active multiplication with acquisition of greater virulence.[1] The effect of solar eclipse on the growth of *S. aureus, Klebsiella* species, and *E. coli* cultures has resulted in phenotypic change [Tables 1–3]. No changes were observed in biochemical reactions, colony characteristics, or capsule formation in case of *Klebsiella* species.

**Table 1:** Comparison of antibiotic susceptibility pattern on MHA : *Staphylococcus aureus*

Antibiotics	Normal sunlight (mm)	Solar eclipse phase (mm)	Standard sensitive zone size (mm)
Penicillin (10 units)	25	24	29	
Cefazolin (30 μg)	27	26	1
Vancomycin (30 μg)	17	16	15
Teicoplanin (30 μg)	11	11	14
Clindamycin (2 μg)	25	24	21
Amoxycillin (20 μg)+ clavulanic acid (10 μg)	27	18	20

**Table 2:** Comparison of antibiotic susceptibility pattern on MHA :*Escherichia coli*

Antibiotics	Normal sunlight (mm)	Solar eclipse phase (mm)	Standard sensitive zone size (mm)
Aztreonam (30 μg)	24	22	22
Amikacin (30 μg)	24	22	17
Piperacillin (100 μg)	24	21	21
Ceftazidime (30 μg)	25	25	18
Ceftazidime (30 μg)+clavulinic acid (10 μg) (ESBL screen)	25	25	25
Ciprofloxacin (5 μg)	40	37	21

**Table 3:** Comparison of antibacterial susceptibility pattern on MHA : *Klebsiella species*

Antibiotics	Normal sunlight (mm)	Solar eclipse phase (mm)	Standard sensitive zone size (mm)
Cefotaxime (30 μg)	31	28	23	
Meropenem (10 μg)	27	25	16
Ciprofloxacin (5 μg)	27	22	21
Piperacillin (100 μg)	18	17	21
Ceftazidime (30 μg)	28	25	18
Amikacin (30 μg)	23	21	17

To observe any mutation on exposure to solar eclipse, a comet assay was performed. The comet assay methodology was performed by layering of normal agarose on slides. Cells are mixed with low-melting agarose and spread over glass microscope slides. Following the lysis of cell membrane and proteins, the unwound DNA subjected to electrophoresis, stained, and image analysis performed. There was no significant change observed in the yeast formed by this assay, which indicates that there may be no major impact of solar eclipse on human beings.

## Discussion

This is an unpublished data stating that solar eclipse does no harm to prokaryotes and eukaryotes. Whatever genotypic and phenotypic variation occurs contributes for the better survival of microbes and man. Further elucidation of impact of solar eclipse studies is to understand the complex nature of the composition of short waves, quantum waves emitted and absorbed in the environment, and the duration of exposure, which decides the phenotypic and genotypic changes in microbes and man.[9,10]

Thus morphologic change contributes to increased pathogenic potential of fungus. This transitional change has also been recorded by molecular studies.[11] There seems to be various environmental stimuli which may enhance a switch between yeast and filamentous growth patterns.[12] The capacity to switch the pattern of growth in response to an environmental change is termed as dimorphism.[7,12,13] Since solar eclipse in India is considered to cause adverse effects on humans, may be to some extent on eyes or skin, the effect of the solar eclipse on the microorganism isolated from clinical isolates was investigated. We also hadevaluated differences in antibiotic sensitivity of microorganisms in comparison with morphological changes within bacterialcells at the time of eclipse and normal sunlight on 15thJanuary, 2010, from 11.15 am to 3.15 pm (total duration = 4 h) to ascertain possibility of mutation as the cause for phenotypic and genotypic change. The phenotypic changes were observed in Gram-stained smear.

## Conclusions

Our observations have important implications of solar eclipse and their induced variations. To our knowledge, such studies have not been reported previously for *S. aureus, Klebsiella* species, *E. coli*, and *C. albicans* in clinical isolates. However from our studies, we could infer that exposure to solar eclipse does help in the favor of microbes and man.[14] Further study is needed in this area to use solar eclipse therapeutically to enhance the immune system and targeting the destruction of genesis of malignant cells thus, to alleviate the suffering of mankind.
